# Vitamin K-Dependent Proteins as Predictors of Valvular Calcifications and Mortality in Hemodialysis Patients

**DOI:** 10.3390/biomedicines13010048

**Published:** 2024-12-28

**Authors:** Marcel Palamar, Iulia Dana Grosu, Adalbert Schiller, Ligia Petrica, Madalina Bodea, Alexandru Sircuta, Elisabeta Gruescu, Oana Daniela Matei, Maria Daniela Tanasescu, Ionut Golet, Flaviu Bob

**Affiliations:** 1 Department of Internal Medicine II—Nephrology University Clinic, “Victor Babeș” University of Medicine and Pharmacy, 300041 Timisoara, Romania; marcel.palamar@umft.com (M.P.); schiller.adalbert@umft.ro (A.S.); petrica.ligia@umft.ro (L.P.); bob.flaviu@umft.ro (F.B.); 2 Centre for Molecular Research in Nephrology and Vascular Disease, Faculty of Medicine, “Victor Babeș” University of Medicine and Pharmacy, 300041 Timisoara, Romania; 3 Deva Emergency County Hospital, 330004 Deva, Romania; elisabetastirbu@yahoo.com (E.G.);; 4 Dialysis, Fresenius Nephrocare Deva, 330004 Deva, Romania; 5 Nephrology, Bucharest Emergency University Hospital, 050098 Bucuresti, Romania; 6 Department of Management, Faculty of Economics and Business Administration, University of the West Timisoara, 300223 Timisoara, Romania

**Keywords:** hemodialysis, calcifications, vitamin K, osteocalcin, matrix Gla protein

## Abstract

**Background/Objectives**: Vitamin K deficiency in chronic kidney disease (CKD) could potentially occur due to multiple factors, leading to an increased risk of vascular and valvular calcifications. Vitamin K status can be indirectly assessed by measuring the blood levels of vitamin K-dependent proteins (VKDPs), such as matrix GLA protein (MGP). This study aims to examine the relationship between the levels of inactive MGP (dp-uc MGP) and the presence of valvular calcifications, as well as its association with mortality in hemodialysis patients. **Methods**: We conducted a single-center study that included 45 CKD G5D patients (hemodialysis for 6 months to 10 years) followed up for 24 months. All patients have been assessed at baseline regarding cardiovascular disease (medical history, echocardiography). Moreover, using standard methods, we determined blood biochemistry, complete blood count, and matrix GLA protein. At 24 months of follow-up, we assessed all-cause mortality and cardiovascular mortality. **Results**: In the studied hemodialysis patients, mean dp-uc MGP was 3285.93 +/− 2092.85 pmol/L. Patients with valvular calcifications had higher levels of dp-uc MGP compared to those without (4521.08 +/− 2263.82 vs. 2487.53 +/− 1446.94 pmol/L, however not statistically significant). The presence and severity of valvular calcifications were significantly associated with the history of treatment with vitamin K antagonists (*p* < 0.05). After 24 months of follow-up, we found an all-cause mortality rate of 24.4%. The level of dp-uc MGP was higher in the group of patients that died (3884.81 +/− 2439.20 vs. 3133.09 +/− 1925.26 pmol/L, *p* > 0.05). Patients with more than one valvular calcification on echocardiography had a significantly higher all-cause mortality risk (*p* = 0.04). In terms of traditional risk factors, we observed an increased risk of all-cause mortality in patients with a history of diabetes mellitus (*p* = 0.02) and aortic stenosis (*p* = 0.01). However, other cardiovascular markers, such as coronary heart disease and ejection fraction < 50%, did not have a statistically significant impact on mortality in our patients. **Conclusions**: In our study, we found that vitamin K deficiency, measured indirectly using the level of VKDP, especially dp-uc MGP, is a predictor of valvular calcifications. Severe valvular calcifications, aortic stenosis, and the presence of diabetes mellitus are risk factors for all-cause mortality in hemodialysis patients.

## 1. Introduction

Chronic kidney disease (CKD) represents an important burden for the public health system nowadays, due to its increasing incidence and association with cardiovascular disease (CVD), which leads to increased mortality. The association with CVD increases in patients with end-stage renal disease, especially patients treated with hemodialysis [1].

One of the main causes leading to cardiovascular complications is represented by calcifications of different cardiovascular structures. Thus, calcifications can occur in different vessels, but also at the level of the heart valves (especially mitral and aortic valves). In patients with CKD, especially in advanced stages, procalcifying factors related to mineral bone disease (CKD-MBD), such as increased parathyroid hormone and serum phosphate, but also uremic toxins and inflammatory biomarkers, trigger and drive valvular calcification. Besides these non-traditional risk factors of CVD, in the pathogenesis of valvular heart disease (VHD), the traditional CVD risk factors, such as advanced age, smoking, arterial hypertension, and the metabolic risk factors such as obesity, dyslipidemia, and diabetes mellitus, are involved as well [2,3].

Among the factors that inhibit vascular and valvular calcifications, an important role is played by vitamin K. Vitamin K is a coenzyme of γ-glutamyl carboxylase, which determines the formation of active vitamin K-dependent proteins (VKDPs) such as matrix Gla protein (MGP) [4,5].

MGP, a protein expressed primarily in the cartilage, heart, and vessels (in the vascular smooth muscle cells—VSMCs), acts as an inhibitor of ectopic soft tissue calcification, playing a protective role against valvular mineralization. MGP prevents calcifications by forming a mass with calcium phosphate, only after the vitamin K-dependent carboxylation of dephosphorylated-uncarboxylated MGP (dp-ucMGP), that is thus transformed into the active “carboxylated” form [6]. The level of inactive forms of VKDP reflects the vitamin K status, high levels being associated with reduced vitamin K [7].

CKD patients, especially those treated with hemodialysis, frequently present a state of vitamin K deficiency due to reduced intake combined with increased consumption or antagonizing treatment, such as vitamin K antagonists [8].

The aim of this study is to correlate the level of inactive MGP (dp-uc MGP) with the presence of valvular calcifications and with mortality in hemodialysis patients.

## 2. Materials and Methods

We conducted a single-center prospective cohort study including 45 CKD G5D patients, all patients undergoing maintenance hemodialysis who were followed up for 24 months, in order to assess the influence of vitamin K deficiency on the mortality of the patients.

### 2.1. Patient Population

The hemodialysis patient group included 45 individuals with CKD G5D, all receiving maintenance hemodialysis at a single center (Fresenius Medical Care Deva, Romania). Out of 138 patients at the center, we included those who provided informed consent and had been undergoing chronic hemodialysis for more than 6 months but less than 10 years (mean dialysis vintage 3.7 ± 2.6 years). Patients scheduled for a kidney transplant, a change in renal replacement therapy, or a transfer to another dialysis center within the next 3 months, as well as those with heart failure and reduced ejection fraction (HFrEF), were excluded. Additionally, patients with a history of infection in the past 6 months were also excluded from the study.

All procedures were in accordance with the ethical standards of the institutional research committee and with the Declaration of Helsinki and approved by the Ethics Committee of VICTOR BABES” UNIVERSITY OF MEDICINE AND PHARMACY, TIMISOARA, ROMANIA (protocol code 34/ 30.06.2021). This study had been authorized by the Dialysis Center’s Ethics Committee. Prior to any study procedure, the eligible patients have been asked to provide a written informed consent.

### 2.2. Clinical and Biochemical Evaluation

Fasting blood samples (after at least 4 h of fasting) were collected from the arteriovenous fistula or central venous catheter just prior to the mid-week hemodialysis session.

The samples were kept at 4 °C for 1 h and then centrifuged at 1000× *g* for 10 min. The resulting serum was stored in aliquots at −80 °C until analysis. The frozen samples were thawed before measurements were taken.

The following parameters were measured: iPTH, calcium, phosphorus, serum albumin, dp-ucMGP, serum C-reactive protein, complete blood count, serum ferritin, sodium, and potassium levels.

To assess matrix GLA protein (dp-ucMGP), a quantitative chemiluminescence method was used with the IDS-iSYS InaKtif MGP (dp-u-MGP) kit from Immunodiagnosticssystems (Bolden, UK), employing Xprep equipment from TE Instruments (Delft, The Netherlands)

Demographic data, including gender and age, as well as medical history (dialysis vintage, CKD etiology, comorbidities like diabetes mellitus, coronary heart disease, stroke, and fractures), were recorded for each patient. Information on medications for CKD-MBD and the use of vitamin K antagonists was also documented.

At the start of the study, as well as during the second and third hours of the dialysis session, electrocardiography and echocardiography (including pulsed Doppler, continuous Doppler, and two-dimensional M-mode) were performed on all patients by the same operator using identical equipment to minimize observer variability. Echocardiography followed the guidelines set by the European Association for Cardiac Imaging (EACI). Left ventricular ejection fraction (LVEF) was calculated using the Simpson method. Heart valve calcification, fibrosis, endocardial calcification, left ventricular end-diastolic and end-systolic diameters (LVTDD and LVTSD), as well as other parameters like the ventricular septum (IVS), left ventricular mass (LVM), aortic atheroma, left atrium (LA), and right ventricle (RV), were assessed.

At the end of the dialysis session, patients’ weight was recorded, and this dry weight value was used to calculate BMI (body mass index).

### 2.3. Follow-Up

The follow-up visit was performed after 24 months and included the assessment of the following parameters: iPTH, calcium, phosphorus, serum albumin, serum C-reactive protein, complete blood count, serum ferritin, serum sodium, and potassium.

The end point of our study was represented by the all-cause mortality at 24 months after inclusion.

### 2.4. Statistical Analysis

Data are presented as the mean ± standard deviation for numerical variables with a Gaussian distribution, median and interquartile range for numerical variables with a non-Gaussian distribution, and as percentages of the subgroup total along with the number of individuals.

Statistical analysis was performed using MedCalc Software, version 12.5.0 (MedCalc, Mariakerke, Belgium). The Kolmogorov–Smirnov test was used to assess the distribution of numerical variables. For comparing qualitative variables, Pearson’s chi-squared test and the Spearman rank correlation test were employed. For comparisons of continuous variables with a normal distribution, analysis of variance (ANOVA) was used, along with the Scheffé test for all pairwise comparisons and Levene’s test for equality of error variances.

To calculate a cut-off for a continuous variable based on survival outcomes, we used Jamovi Project (2022), version 2.3 [Computer Software], available at https://www.jamovi.org, accessed 12 December 2022. Once the cut-off was determined, median survival times and 1-, 3-, and 5-year survival rates were calculated. Data were organized using quartiles, which served as cut-off points between each group.

For statistical computing and graphics, R Core Team (2021) R: A Language and Environment for Statistical Computing (Version 4.1) [Computer Software] was used. The log-rank, Gehan, Tarone–Ware, and Peto–Peto tests were applied for comparing differences in survival analysis.

## 3. Results

The study enrolled 45 patients on maintenance hemodialysis, with a mean age of 64.3 ± 10.8 years, 19 female and 26 male patients. Mean BMI at inclusion was 28.08 ± 4.4 kg/m^2^ with a mean abdominal circumference of 115.89 ± 18.12 cm. The biological characteristics of the studied lot at baseline and follow-up are presented in Table 1.

We obtained mean levels of the VKDP (dp-uc MGP) in our patients at baseline of 3285.93 ± 2092.85 pmol/L. The dp-uc MGP normal levels according to the literature are <300–532 pmol/L for the same type of assay used in our study [9].

There were no statistically significant differences in the levels of VKDP between patients with and without a history of various comorbidities, including diabetes mellitus (13/45), coronary heart disease (18/45), or stroke (5/45).

Left ventricular hypertrophy was present in 71.11% (32/45) of patients upon echocardiography. Using ANOVA, we found that there was no difference regarding dp-uc MGP between patients with and without left ventricular hypertrophy (LVH). There was no statistically significant correlation between dp-uc MGP and the ejection fraction; however, patients with ejection fractions lower than 40% have been excluded from the study.

**Figure 1:**
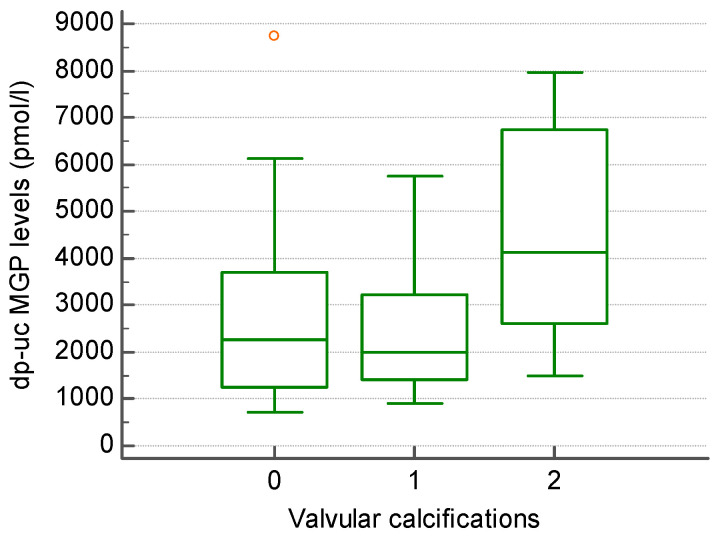
The level of dp-uc MGP in patients without (0), with one (1) and more than one (2) valvular calcification present in echocardiography.

Regarding VHD, valvular calcifications were present in 26/45 patients (55.55%), with 13 patients having one calcification, while 12 patients had more than one calcification. Patients with valvular calcifications (more than one calcification) had higher levels of dp-uc MGP compared to those without (4521.08 +/− 2263.82 vs. 2487.53 +/− 1446.94 pmol/L; however, not statistically significant) (Figure 1).

**Figure 2:**
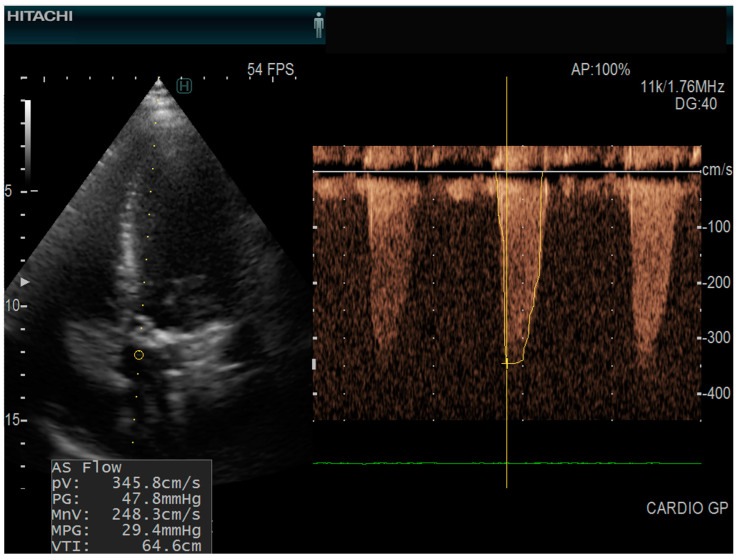
Echocardiography of a patient with aortic stenosis echocardiograph: Hitachi Arietta 65, apical five chamber view, continuous pulse wave doppler AS Flow = aortic stenosis flow; (PV = peak velocity; PG = peak gradient; MnV = mean velocity; MPG = mean pressure gradient; VTI = velocity time integral).

The types of valvular diseases are mentioned in Table 2. In our group, 7/45 patients (15.5%) had aortic stenosis (Figure 2). Patients with aortic stenosis had higher levels of dp-uc MGP; however, not statistically significant (4488.7 +/− 3244.97 vs. 2995.43 +/− 1791.58 pmol/L). The level of dp-uc MGP showed a weak direct statistically significant correlation with aortic flow speed (R = 0.3; *p* = 0.05). We could not find any statistically significant relationship between other cardiovascular disease markers (mitral, pulmonary, or tricuspid valvular parameters) and the level of dp-uc MGP.

Thirteen out of forty-five patients were undergoing treatment with acenocoumarol (vitamin K antagonist), and in this subgroup of patients, dp-uc MGP level was significantly higher (5693.0 ± 1728.64 vs. 2276.5 ± 1232.54 pmol/L, *p* < 0.01). The presence and severity of valvular calcifications were statistically significantly associated with the history of treatment with vitamin K antagonists (*p* < 0.05).

### Survival Analysis

**Figure 3:**
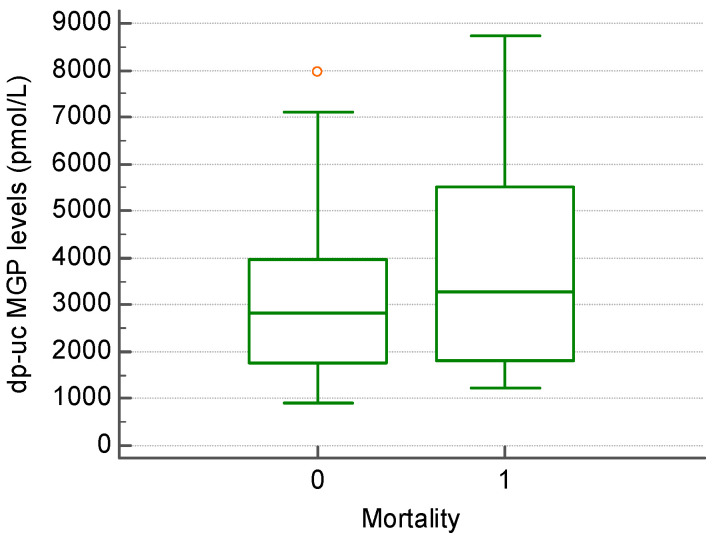
The level of dp-uc MGP in patients that showed a 24-month survival (0) compared to those that died within 24 months of follow-up (1).

For the patient follow-up, we used 24-month all-cause mortality as the endpoint. After 24 months, the mortality rate was found to be 24.4% (Figure 2). Of the deaths, 6 patients (54.5%) died from cardiovascular causes, 4 patients (36.3%) from infections, and 1 patient from upper gastrointestinal bleeding. Mortality was calculated from the time of study inclusion. Among this group, the mortality rate after 5 years of hemodialysis was 20.9%. The level of dp-uc MGP was higher in the group of patients that died, the difference not reaching statistical significance (3884.81 +/− 2439.20 vs. 3133.09 +/− 1925.26 pmol/L, *p* > 0.05) (Figure 3).

**Figure 4:**
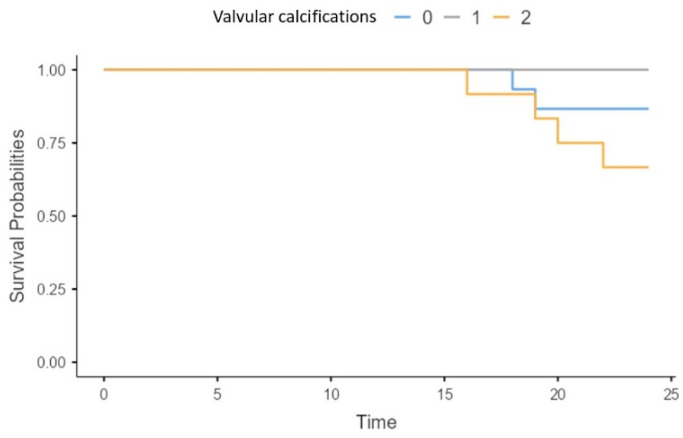
Survival of patients without (0), with one (1) and more than one (2) valvular calcification present in echocardiography.

Patients with more than one valvular calcification on echocardiography had a significantly higher all-cause mortality risk (X2 = 5.58; *p* = 0.06) (Figure 4).

**Figure 5:**
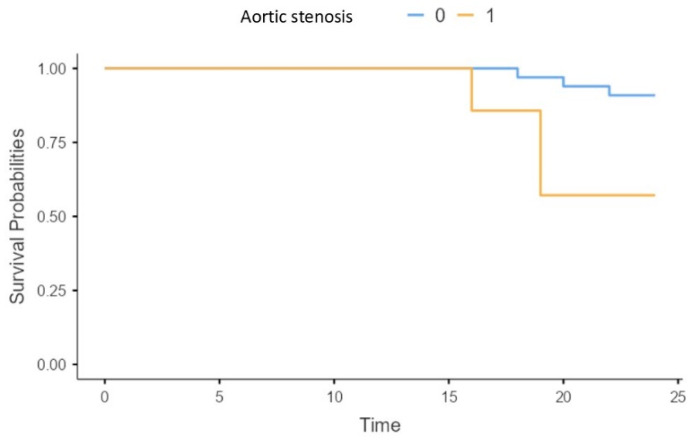
Survival of patients without (0), and with (1) aortic stenosis.

The presence of aortic stenosis was associated with a significantly higher risk of mortality in our patients (X2 = 6.19; *p* = 0.013) (Figure 5).

**Figure 6:**
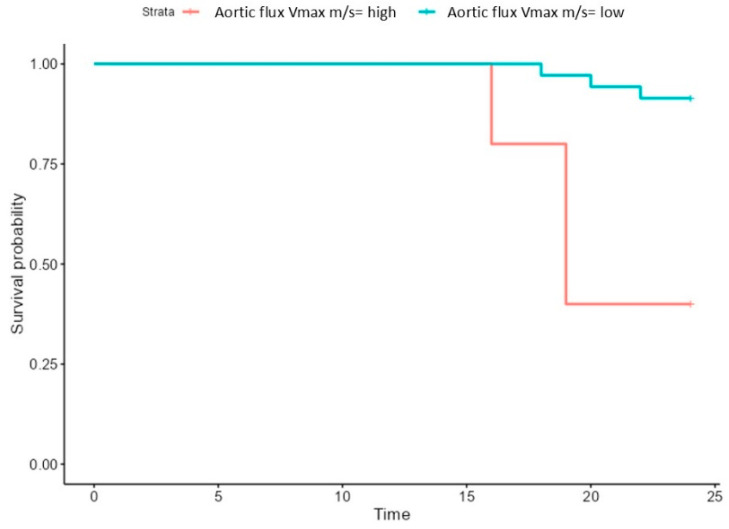
Survival of patients with high (>2.6 m/s) and low aortic (<2.6 m/s) flux.

The risk of mortality was associated with an increased aortic flow, mortality being significantly increased in patients with aortic flow higher than a cut-off value of 2.6 m/s (Figure 6).

Other cardiovascular markers, such as coronary heart disease and ejection fraction < 50%, did not have a significant impact on mortality in our patients.

**Figure 7:**
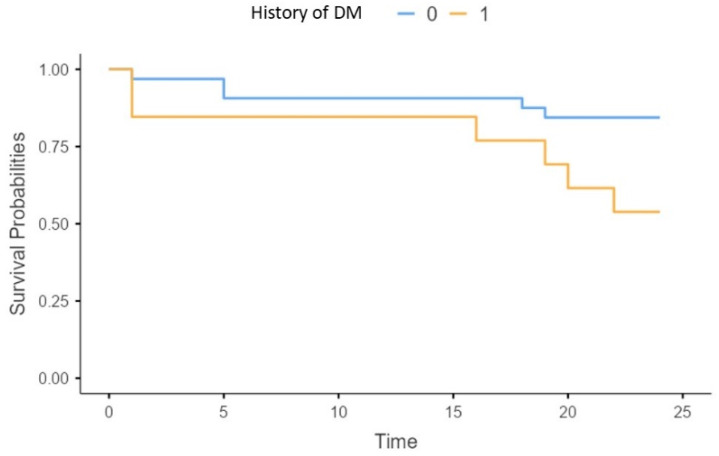
Survival of patients without (0), or with diabetes mellitus (1).

Concerning traditional risk factors, we found a higher risk of all-cause mortality in patients with a history of diabetes mellitus (X2 = 4.5; *p* = 0.03) (*p* = 0.02) (Figure 7).

## 4. Discussion

The presence of valvular calcifications is associated with an increased cardiovascular morbidity and mortality in patients with CKD [10]. Cardiovascular mortality accounts for almost half of the deaths of end-stage renal disease patients, with one of the main culprits being extensive extraosseous calcifications [11]. Besides atherosclerosis, vascular calcifications represent the underlying pathophysiologic mechanism responsible for cardiovascular events in dialysis patients [12].

Hence, the reason for investigating pathways related to the calcification burden is of particular clinical interest.

In end-stage renal disease (ESRD) patients on chronic HD therapy, vitamin K deficiency is present, especially due to low intake, more than due to HD wash out [13]. Vitamin K has a role in reducing the level of vascular and valvular calcifications through the activation of vitamin K-dependent proteins, such as MGP.

As mentioned in the introduction, vitamin K leads to the activation of the undercarboxylated form of MGP, the result being the reduction in calcium precipitation in the vessels. Decreased levels of vitamin K are associated with high levels of dp-uc MGP. A longitudinal study has proven that serum dp-uc MGP increases alongside the decrease in eGFR in a cohort followed up for 8.9 years [14]. In our patients, the level of dp-uc MGP was higher than compared to normal ranges.

Despite the fact that in our patients there was no relationship between the history of cardiovascular events (stroke or coronary heart disease) and the level of VKDP, the presence of VHD is associated with serum dp-uc MGP level. We found higher (not statistically significant) levels of dp-uc MGP in patients with valvular calcifications or in those with aortic stenosis, and also a correlation with aortic flow speed.

In addition to vascular calcifications, in CKD patients, previous studies have shown that the level of MGP is associated with the presence of valvular calcification and consequently to the increase in cardiovascular and also all-cause mortality [15,16,17].

Among valvular diseases, calcific aortic stenosis is the most common one, having no available pharmacologic treatment. The development of calcific aortic stenosis is associated with cardiovascular risk markers such as arterial hypertension, hypercholesterolemia, diabetes mellitus (DM), male gender, cigarette smoking, or older age [18]. In CKD there is an increased risk of calcific aortic stenosis, especially in patients treated with hemodialysis [19]. The prevalence of severe aortic stenosis ranges from 4% to 13%, with more than half of these patients having low-flow, low-gradient severe aortic stenosis [2]. In our group of patients, the prevalence of aortic stenosis was 15.5%. Moreover, there is a proven association between the presence of aortic valve calcifications and a lower ejection fraction, worse diastolic function, and increased pulmonary hypertension in a cohort of patients including both CKD and non-CKD patients [20].

In the ASTRONOMER study, a faster progression of aortic valve calcifications was associated with higher levels of total dpMGP after a follow-up period of 3.5 years [21]. Similar findings were reported by Ueland et al., who found not only an association between high levels of dp-ucMGP and symptomatic aortic sclerosis but also a 9-fold increased all-cause mortality in patients with high dp-uc MGP [22].

Previous data have identified positive correlations of the elements of CKD-MBD-PTH [23], calcium, and phosphorus with vitamin K deficiency (expressed through dp-uc MGP) and subsequently with aortic stenoses. In our patients, however, there is a closer relationship between valvular calcifications and vitamin K status than with elements of CKD-MBD, and as shown in a previous study, the direct link between vitamin K deficiency and CKD-MBD could not be proved [24]. In addition, the link between CKD-MBD and aortic stenosis was also not present; in our patients, there was no statistically significant correlation between aortic stenosis and PTH, serum calcium, or serum phosphorous.

As already mentioned above, the relationship between aortic stenosis and dp-uc MGP seems to be slightly more evident, with increased levels of VKDP in patients with aortic stenosis (not statistically significant), but with a weak statistically significant correlation between the aortic flow and MGP.

Regarding the survival analysis, increased peak aortic valve jet velocity (Vmax) was associated with a higher mortality, confirmed also by the fact that the presence of aortic stenosis leads to a lower survival in our patients. Similar results have been obtained in a study performed in patients undergoing open surgery for chronic limb threatening ischemia; Vmax was a significant independent predictor of all-cause death within 2 years after the procedure in dialysis patients, but not in patients managed without dialysis [25].

The presence of valvular calcifications overall was associated with a higher mortality in our hemodialysis patients. This aspect has been shown in other studies as well, and even in early stages of CKD, the presence of cardiovascular calcifications leads to a higher mortality rate, especially in patients with diabetes mellitus. Other available data show a positive correlation between dp-ucMGP and diabetes history [26] and also with BMI [9] or visceral fat index [27]. However, despite the relationship between diabetes mellitus and mortality in our patients, there was no direct association between the level of VKDP and diabetic status.

There was, however, an interesting association between higher dp-ucMGP and inflammation. The available literature data regarding this association are scarce and do not involve patients with CKD. In a presentation by Schweighofer et al. [28], dp-uc MGP was significantly higher in persons with increased CRP and ferritin levels. In systemic sclerosis patients, an immune-mediated condition, dp-ucMGP predicted a higher risk of 20-year cardiovascular death, independent of traditional risk factors [29]. Our finding may suggest a possible link between inflammation and vitamin K status in HD patients, who are known for their persistent pro-inflammatory state.

Another factor leading to vitamin K deficiency, and to the presence of valvular calcifications in our patients, was the treatment with vitamin K antagonists (VKAs) (acenocoumarol). The anticoagulant effects of acenocoumarol are mediated through the inhibition of vitamin K epoxide reductase, which impairs activation of coagulation factors. It has been shown in other studies as well that the treatment with VKA worsens vitamin K deficiency, proven by the higher levels of MGP [30], but also the association between VKA and valvular (especially aortic valve) calcifications [31].

The importance of assessing valvular heart disease, especially in hemodialysis patients, is a consequence of the fact that it has no effective medical therapy [32].

It is clear that CKD patients with severe aortic stenosis who receive conservative treatment have a poor prognosis, experiencing significantly higher mortality rates compared to those who undergo valve replacement [33]. These procedures are, however, associated with increased incidence of complications and high costs.

It has been proven that dp-ucMGP can reflect the vascular vitamin K status. Moreover, it could serve as a biomarker of cardiovascular health and potentially as a marker of mortality in CKD patients [34].

The major limitations of our study are that it is a cross-sectional analysis, single-centered, and has a small lot of subjects. Moreover, we have not included in our analysis other possible confounding factors for high levels of dp-ucMGP, such as HbA1c or lipid profile, and we excluded patients with HFrEF.

## 5. Conclusions

The main conclusion is that vitamin K deficiency, measured indirectly using the level of VKDP-dp-uc MGP, is a predictor of valvular calcifications in HD patients and is associated with higher levels of inflammation. Severe valvular calcifications, aortic stenosis, and the presence of diabetes mellitus are risk factors for all-cause mortality in hemodialysis patients.

Our findings highlight the potential perspective of using VKDP (especially dp-uc MGP) as a measure of vitamin K deficiency and as a marker of cardiovascular disease, especially valvular heart disease in HD patients.

More extensive studies are needed to validate these statements.

## Figures and Tables

**Table 1:** Characteristics of the studied lot (the results are expressed as mean values ± standard deviations).

Variable	Mean Values ± Standard Deviations at Baseline	Mean Values ± Standard Deviations at Follow-Up
Serum albumin (g/dL)	3.66 ± 0.2792	3.8 ± 0.4
Hemoglobin (g/dL)	11.09 ± 1.183	10.6 ± 1.4
Hematocrit (%)	33.76 ± 3.74	32.2 ± 4.3
Serum ferritin (ng/mL)	529.4 ± 530.6	455.5 ± 477.4
Serum calcium (mg/dL)	9.18 ± 0.82	9.1 ± 0.6
Serum phosphorus (mg/dL)	5.63 ± 1.41	5.2 ± 1.4
iPTH (pg/mL)	198.12 ± 130.1	289.6 ± 262.2
Bicarbonate (mEq/L)	21.46 ± 2.578	21.18 ± 2.26
Predialysis K (mmol/L)	5.47 ± 1	4.9 ± 0.8
CRP (mg/dL)	14.71 ± 15.34	15.8 ± 38.1

No statistically significant correlation was found between the studied biological parameters at baseline and dp-uc MGP, except with markers of inflammation (CRP) (r = 0.55, *p* = 0.004).

**Table 2:** Patients with different valvular diseases.

Parameter (at Baseline)	Number of Patients (out of 45)	Percentage
Mitral stenosis	25	55.55%
Mitral insufficiency	37	82.22%
Aortic stenosis	7	16%
Aortic insufficiency	22	49%
Tricuspid stenosis	0	0
Tricuspid insufficiency	38	84.44%
Pulmonary stenosis	0	0
Pulmonary insufficiency	1	2%

## Data Availability

The original contributions presented in this study are included in the article. Further inquiries can be directed to the corresponding author.
